# Education and Lifestyle Factors Are Associated with DNA Methylation Clocks in Older African Americans

**DOI:** 10.3390/ijerph16173141

**Published:** 2019-08-28

**Authors:** Wei Zhao, Farah Ammous, Scott Ratliff, Jiaxuan Liu, Miao Yu, Thomas H. Mosley, Sharon L. R. Kardia, Jennifer A. Smith

**Affiliations:** 1 Department of Epidemiology, School of Public Health, University of Michigan, Ann Arbor, MI 48109, USA; 2 Department of Epidemiology, Harvard T.H. Chan School of Public Health, Boston, MA 02115, USA; 3 Memory Impairment and Neurodegenerative Dementia (MIND) Center, University of Mississippi Medical Center, Jackson, MS 39126, USA; 4 Survey Research Center, Institute for Social Research, University of Michigan, Ann Arbor, MI 48104, USA

**Keywords:** DNA methylation, epigenetic age, education, lifestyle risk factors, African American, GENOA

## Abstract

DNA methylation (DNAm) clocks are important biomarkers of cellular aging and are associated with a variety of age-related chronic diseases and all-cause mortality. Examining the relationship between education and lifestyle risk factors for age-related diseases and multiple DNAm clocks can increase the understanding of how risk factors contribute to aging at the cellular level. This study explored the association between education or lifestyle risk factors for age-related diseases and the acceleration of four DNAm clocks, including intrinsic (IEAA) and extrinsic epigenetic age acceleration (EEAA), PhenoAge acceleration (PhenoAA), and GrimAge acceleration (GrimAA) in the African American participants of the Genetic Epidemiology Network of Arteriopathy. We performed both cross-sectional and longitudinal analyses. In cross-sectional analyses, gender, education, BMI, smoking, and alcohol consumption were all independently associated with GrimAA, whereas only some of them were associated with other clocks. The effect of smoking and education on GrimAA varied by gender. Longitudinal analyses suggest that age and BMI continued to increase GrimAA, and that age and current smoking continued to increase PhenoAA after controlling DNAm clocks at baseline. In conclusion, education and common lifestyle risk factors were associated with multiple DNAm clocks. However, the association with each risk factor varied by clock, which suggests that different clocks may capture adverse effects from different environmental stimuli.

## 1. Introduction

DNA methylation is an epigenetic mechanism that plays an important role in regulating gene expression without changes in the DNA sequence. Prior studies suggest that DNA methylation is modifiable throughout the life course and that methylation levels at many sites correlate with chronological age [1]. Therefore, it is considered an internal biomarker of chronological age, which is more representative of the physiological age of the human body. To capture these properties of DNA methylation, methylation clocks (DNAm Age), based on aggregated DNA methylation levels at selected CpG sites, have been developed [2]. The earlier generation of DNAm clocks aimed to predict chronological age with the assumption that chronological age is a good surrogate of biological age. The most representative and successful two DNAm Age estimators were independently developed by Horvath and Hannum in 2013 [3,4]. Hannum’s DNAm Age (herein referred to as HannumAge) is a blood-specific clock whereas Horvath’s DNAm Age (herein referred to as HorvathAge) is a clock that is robust across multiple tissues/cells. Later, Levine, et al. developed a new blood-based clock (herein referred to as PhenoAge), which is a biomarker trained on many aging-related clinical measurements, including albumin, creatinine, glucose, C-reactive protein, alkaline phosphatase, white blood cell count, and other metrics [5]. One of the most recent additions is the GrimAge clock, which combines DNAm surrogates of seven plasma proteins and smoking along with gender and age [6].

DNAm Age acceleration, the residual from fitting DNAm Age to chronological age, is hypothesized to reflect the cellular aging of a person’s body relative to their chronological age. Consistent with this hypothesis, the intrinsic epigenetic age acceleration (IEAA) derived from HorvathAge, the extrinsic epigenetic age acceleration (EEAA) derived from HannumAge, PhenoAge acceleration (PhenoAA) derived from PhenoAge, and GrimAge acceleration (GrimAA) derived from GrimAge, have been associated with a variety of age-related diseases including cancer, cardiovascular, and all-cause mortality in multiple populations [5,6,7,8,9,10,11,12,13,14,15]. All of these suggest that DNAm clocks plays a role in the process of aging and are good biomarkers of cellular aging.

Earlier studies have demonstrated that DNA methylation is influenced by environmental factors and may reflect the cumulative burden of adverse effects from the environment throughout the life course [16]. It may be a mechanism through which external stimuli get imbedded into the body to influence aging and disease susceptibility. Therefore, there has been increasing interest in identifying external environmental and/or lifestyle risk factors that influence DNAm clocks. Education, in particular, is an important social determinant of health, since low educational attainment increases susceptibility for many age-related chronic diseases [17,18,19]. Moreover, low education is often correlated with many lifestyle risk factors that are known to influence healthy aging, like smoking, heavy alcohol consumption, low physical activity, and higher BMI. Therefore, it is now critical to evaluate the association between education as well as lifestyle risk factors with DNAm clocks with the aim of identifying modifiable risk factors of aging and gaining a deeper understanding of the potential underlying cellular mechanisms.

To date, the relationship between education and lifestyle risk factors and the aforementioned DNAm clocks have been examined in various contexts [5,20]. However, the association between these factors and GrimAA, which has been shown to have better performance predicting lifespan and health, has not yet been carefully explored [6]. Moreover, most studies on DNAm clocks were conducted largely on post-menopausal women. It is well known that human aging patterns differ by gender, as women have a longer lifespan then men. An earlier study also suggested that the environment interacts with gender to shape the human methylome [21]. Thus, gender is an important factor to consider when the contribution of environment to methylome aging is examined. A very recent study examined the association between education and lifestyle risk factors with a number of DNAm clocks in a large-scale gender balanced cohort. However, the study participants were of European ancestry [22]. Given that the aging process varies by gender and ethnicity [23,24], representative studies are needed to draw appropriate conclusions. To the best of our knowledge, no previous study has examined the association between education and lifestyle risk factors with DNAm clocks in a gender balanced, adequately powered, African-American cohort or considered interaction between lifestyle factors and gender.

To fill this gap, the aim of this study is to assess the effects of gender, BMI, education, smoking, alcohol consumption, and physical activity on four important DNAm clocks in African-American participants from the Genetic Epidemiology Network of Arteriopathy (GENOA). We hypothesized that low education and all lifestyle risk factors for healthy aging would be associated with DNAm age acceleration. Most of the prior studies exploring the association between lifestyle factors and DNAm Age have been cross-sectional; however, longitudinal analysis would allow exploration of temporal relationships between lifestyle factors and DNAm clocks and provide unique insight regarding causality. Thus, we also utilized a subset of participants whose methylation data was measured at two time points to assess whether any of the risk factors are associated with longitudinal changes in DNAm Age acceleration.

## 2. Materials and Methods

### 2.1. Study Sample

The Genetic Epidemiology Network of Arteriopathy (GENOA) study is a community-based study that was designed to examine the genetic effects of hypertension and related target organ damage. The study includes both African-Americans (AA) from Jackson, Mississippi and European Americans (EA) from Rochester, Minnesota [25]. In the initial phase of GENOA (Phase 1: 1996−2001), all members of the sibships containing at least two individuals who were clinically diagnosed with hypertension prior to age 60 were recruited. Exclusion criteria included secondary hypertension, alcoholism or drug abuse, pregnancy, insulin-dependent diabetes mellitus, or active malignancy. In the second phase (Phase 2: 2001−2005), all participants were invited for a second examination. Eighty percent of AAs (N = 1482) and 75% of EAs (N = 1213) from Phase 1 returned. Demographic information, medical history, clinical characteristics, lifestyle factors, and blood samples were collected in each phase. Written informed consent was obtained from all subjects and approval was granted by participating institutional review boards (University of Michigan, University of Mississippi Medical Center, and Mayo Clinic). Methylation data was collected from AA participants only.

### 2.2. Methylation Measures

Genomic DNA was extracted from stored peripheral blood leukocytes that were collected at Phase 1 (N = 1106) or Phase 2 (N = 304) using an AutoGen FlexStar (AutoGen, Holliston, MA, USA). Bisulfite conversion was performed with the EZ DNA Methylation Kit (Zymo Research, Irvine, CA, USA), and methylation was assessed using the Illumina Infinium HumanMethylationEPIC BeadChip. After obtaining the raw intensity data, we first used the shinyMethyl R package to generate the density plot to identify sex mismatch or sample outliers [26]. We further checked the sample identity using 59 SNP probes implemented in the EPIC chip and removed mismatched samples. Lastly, we removed samples with incomplete bisulfite conversion identified using the QCinfo function in the R package [27]. The Minfi R package was used to perform background correction and normalization using ssNoob [28]. The regression on correlated probes (RCP) method was used to adjust probe-type bias [29]. Individual probe measurements with detection *p*-value < 1 × 10^−16^ were considered successfully detected. Probes with more than 10% undetected samples were excluded [30]. After quality control, a total of 1396 samples (N = 1100 with Phase 1 data; N = 296 with Phase 2 data; N = 293 with both Phase 1 and Phase 2 data) and 857,121 CpG sites remained for analyses.

### 2.3. DNAm Age Calculation and Blood Cell Counts

Methylation beta values were uploaded to the new online Horvath epigenetic age calculator to calculate DNAm Age [31]. HannumAge was estimated using 71 CpG sites as specified in Hannum et al., 2013 [3,4]. EEAA is the residual from a regression model that regresses HannumAge aggregated with three blood cell components (naïve cytotoxic T cells, exhausted cytotoxic T cells, and plasmablasts) on chronological age. It captures both intrinsic DNAm age as well as age-related changes in blood cell composition. HorvathAge was estimated using 353 CpGs as specified in Horvath, et al., 2013 [4]. IEAA is the residual from the regression model that regresses HorvathAge on chronological age and blood cell counts. Thus, it captures cell-intrinsic properties of aging that are independent of cell types. PhenoAge was estimated using 513 CpG sites [5], and GrimAge was estimated using 1030 CpG sites [6]. GrimAA and PhenoAA are the residual of the regression models that regress GrimAge or PhenoAge on chronological age without adjusting for blood cell counts. Lastly, GrimAge was developed as a combination of DNAm surrogates of eight individual items, including seven plasma proteins (adrenomedullin (ADM), beta-2 microglobulin (B2M), growth differentiation factor 15 (GDF15), Cystatin C (CystatinC), leptin (Leptin), plasminogen activation inhibitor 1 (PAI1), tissue inhibitor metalloproteinase 1 (TIMP1)) and the amount of cigarettes smoked (PACKYRS). For example, the DNAmGDF15 was calculated using 137 CpG sites and is an estimate of the amount of protein GDF15 in the plasma. To better understand GrimAge, we also obtained the DNAm surrogates of all eight individual items from the online calculator. We note that the EPIC array does not include all of the CpG sites that were originally used to construct the HorvathAge (EPIC is missing 19 sites) and HannumAge (EPIC is missing 6 sites). However, previous studies have demonstrated that this does not substantively compromise the performance of the DNAm age predictors, especially the age acceleration measurements [32,33]. The proportion of blood cell counts, including CD8+ T, CD4+ T, natural killer, B cells, and granulocytes were estimated using Houseman’s method as implemented in the online epigenetic age calculator [34].

### 2.4. Assessment of Education, Lifestyle Factors and Other Covariates

Educational attainment was classified based on the self-reported highest degree obtained as well as years of education at Phase 1. Participants were classified as having less than a high school degree (<12 years of education), high school (HS) degree or equivalent (12 years or General Education Development (GED)), and some college and above (>12 years). Self-reported smoking status was assessed at both Phase 1 and Phase 2. Current smokers were defined as the people who reported smoking at the time of exam and had smoked at least 100 cigarettes in his or her lifetime. Former smokers were defined as those who had smoked at least 100 cigarettes in his or her lifetime but do not currently smoke. Never smokers were individuals who had not smoked more than 100 cigarettes in his or her lifetime. Alcohol consumption was calculated as the number of drinks per week based on aggregated measurements of a variety of alcoholic drinks. Due to the skewed distribution of alcohol consumption, this variable was log transformed prior to analyses. A reliable measurement of physical activity was not assessed in Phase 1 of GENOA. Since people usually do not change their physical activity dramatically, the physical activity assessed at Phase 2 was used in this study with the assumption that physical activity at Phase 1 and Phase 2 would be highly correlated. It was calculated as a weighted sum of the average number of hours per day spent on moderate (weight = 1) and vigorous (weight = 2) physical activity. Gender, age and BMI were also collected at both phases. Body mass index (BMI, kg/m^2^) was calculated from measured weight and height.

### 2.5. Statistical Analyses

First, we performed cross sectional analyses to assess the association between education, smoking, alcohol consumption, physical activity, BMI, and gender with DNAm Age acceleration at Phase 1. For each DNAm Age acceleration measure and each risk factor, we built a linear mixed model with DNAm Age acceleration as the outcome and the variable of interest as the predictor adjusting for age, gender, and familial relationship as a random effect (Model 1, minimally adjusted model). To assess the unique contribution of each variable after controlling for all others, we also built multivariable models with each DNAm Age acceleration measure as the outcome and all the associated variables of interest as the predictors along with age and gender (Model 2, fully-adjusted model). If a DNAm Age acceleration measure was associated with both gender and a lifestyle factor, we further assessed whether the observed effect of the lifestyle factor on DNAm Age acceleration differed by gender by adding the interaction term of gender and the corresponding variable (Model 3, interaction model). For any significant interaction, we formally assessed the gender-specific effect by performing contrast tests. Lastly, since GrimAge was comprised of DNAm surrogates of eight individual items as described before, for any lifestyle factors that were associated with GrimAA, we further assessed their association with each component to identify those that may drive the association (Model 2, fully adjusted model). GENOA is a predominately hypertensive cohort, and hypertension status could be a potential confounding factor. Therefore, we ran Model 2 on a subgroup of people who had hypertension as a sensitivity analysis. Since both GrimAA and PhenoAA are correlated with blood cell count, we also ran sensitivity analysis for any models that involved GrimAA, PhenoAA, and GrimAge components by additionally adjusting for proportions of blood cell counts to evaluate if any of the associations were due to a difference in blood cell composition.

We also performed longitudinal analyses on a subset of samples whose DNA methylation was measured at both Phase 1 and Phase 2. Since physical activity did not show association with any DNAm clock and it was assessed at Phase 2 only, we did not examine this variable in longitudinal analyses. We built fully adjusted linear mixed models with DNAm Age acceleration at Phase 2 as the outcome and age, gender, education, BMI, smoking, and alcohol consumption at Phase 1 as predictors. The model additionally adjusted for DNAm Age acceleration at Phase 1 and any difference between Phase 1 and Phase 2 (time, BMI difference, and alcohol consumption) that may cause a change in methylation (Model 4, longitudinal model). Familial relationship was adjusted as a random effect. In longitudinal analyses, we were also interested in the effect of age as a predictor. There were 27 participants whose smoking status changed from Phase 1 to Phase 2. Since smoking has a dramatic effect on DNA methylation which may severely confound the results, we only included the participants whose smoking status stayed the same for longitudinal analyses (N = 266). Similarly, we performed sensitivity analysis for GrimAA and PhenoAA by additionally adjusting for changes in blood cell counts to assess whether the association we observed was independent of the changes in blood cell composition.

## 3. Results

### 3.1. Descriptive Statistics

The descriptive statistics of the study sample at Phase 1 are presented in Table 1. The GENOA participants had an average age of 57 (±10.5) years at Phase 1, and 29% of them were male. About 39% of the participants had at least some college education, 27% had a high school or equivalent degree, and the remainder had less than a high school degree. The participants tended to be overweight with an average BMI of 31 (±6.5) kg/m^2^, and they drank an average of 0.66 (±2.7) drinks per week. A majority of the participants never smoked, whereas 23% and 16% of the participants were former and current smokers, respectively. The estimated DNAm Age tended to be lower than chronological age and varied by clock with GrimAge being the highest (mean = 54 years) and PhenoAge being the lowest (mean = 44 years).

The descriptive statistics of the study sample that were used for longitudinal analyses are also presented in Table 1. The participants at the Phase 2 exam were on average 5.4 years older than they were at the Phase 1 exam (mean age is 54.0 years at Phase 1 and 59.4 years at Phase 2). In comparison, the differences of DNAmAge between the two phases is 3.9 years for HorvathAge, 4.8 years for HannumAge, 4.5 years for PhenoAge, and 3.4 years for GrimAge. Most of the lifestyle risk factors were correlated between Phase 1 and Phase 2 with similar means except for BMI which increased by 0.5 kg/m^2^ from 31.5 at Phase 1 to 32.0 at Phase 2 (*p* < 0.001).

### 3.2. Correlation among DNAm Age Estimators

The scatterplots of DNAm Age estimators against chronological age at Phase 1 are shown in Figure S1. As expected, chronological age is significantly correlated with HorvathAge (r = 0.86), HannumAge (r = 0.90), PhenoAge (r = 0.82), and GrimAge (r = 0.85; all *p* < 0.0001). The correlations are comparable to those in other studies and validate the utility of those estimators in this study. The pairwise correlation among the four DNAmAge estimators ranges from 0.77 between GrimAge and HorvathAge to 0.90 between HannumAge and HorvathAge (Table S1). However, the correlation among the DNAmAge acceleration measurements (after adjusting for age) are much lower and range from 0.19 (IEAA and GrimAA) to 0.50 (EEAA and PhenoAA).

We also assessed the correlation of DNAm Age at Phase 1 vs. Phase 2 for the set of people with DNAm Age estimated at both time points (Table 1). All DNAm clocks at Phase 1 and Phase 2 were highly correlated, with the lowest correlation observed for HorvathAge (r = 0.919) and highest correlation for GrimAge (r = 0.972). Similarly, the DNAm Age acceleration measurements at Phase 1 and Phase 2 were also correlated, but to a lesser extent, with the lowest correlation observed for IEAA (r = 0.748) and highest correlation observed for GrimAA (r = 0.911).

### 3.3. Associations between Education or Lifestyle Risk Factors and DNAm Age Acceleration

We examined the association between each risk factor (gender, education, smoking, alcohol consumption, physical activity, and BMI) with each DNAm Age acceleration measure one at a time using linear mixed models adjusting for age, gender and familial relationship (random effect). As shown in Table S2, male gender is strongly positively associated with GrimAA (*p* = 1.69 × 10^−35^), IEAA (*p* = 2.16 × 10^−5^) and EEAA (*p* = 1.26 × 10^−10^), but not with PhenoAA (*p* = 0.647). Higher education, especially some college education, was significantly associated with lower age acceleration for all clocks (GrimAA, *p* = 7.56 × 10^−7^; PhenoAA, *p* = 0.004; IEAA, *p* = 0.018; EEAA, *p* = 2.19 × 10^−5^). High school education, to a lesser extent, was also associated with lower IEAA (*p* = 0.049) and EEAA (*p* = 0.019) and was marginally associated with GrimAA (*p* = 0.060) and PhenoAA (*p* = 0.065). These associations suggest a dose-dependent effect of educational attainment on DNAm Age acceleration. Similarly, smoking showed a dose-dependent effect with GrimAA and PhenoAA, with current smoking conferring a larger effect on DNAm Age acceleration than former smoking. However, the dose response was not present for IEAA, which was only associated with former smoking (*p* = 0.009), and smoking was not associated with EEAA. Alcohol consumption was associated with higher GrimAA (*p* = 2.76 × 10^−9^) and PhenoAA (*p* = 0.038), but not IEAA or EEAA. The direction of effect for all observed associations was as expected. In particular, higher education was associated with younger DNAm Age relative to chronological age; smoking, alcohol consumption, and higher BMI were all associated with older DNAm Age relative to chronological age; and even though physical activity was not significantly associated with any DNAm Age measurement, the direction of effect was negative which is consistent with our hypothesis.

In Model 2, we first fit a single multivariable model with all lifestyle factors (including physical activity) to assess the unique contribution of each predictor to the outcome of interest. Since physical activity did not show any association with the acceleration of any DNAm Age estimator (Table S3) and it was not assessed concurrently with other lifestyle factors, we decided to drop it from Model 2. As shown in Table 2, after adjusting for all other lifestyle factors, gender (*p* = 1.94 × 10^−17^), some college education (*p* = 0.041), smoking (*p* = 2.67 × 10^−15^ for former smoker, *p* = 4.98 × 10^−81^) for current smoker), alcohol consumption (*p* = 0.033), and BMI (*p* = 0.029) were all independently associated with GrimAA. Only smoking (*p* = 0.015 for former smoker, *p* = 0.001 for current smoker) and BMI (*p* = 0.022) were associated with PhenoAA. Gender (*p* = 0.003), some college education (*p* = 0.050) and former smoking (*p* = 0.009) were independently associated with IEAA, whereas gender (*p* = 1.49 × 10^−8^) and education (*p* = 0.028 for high school education, *p* = 1.02 × 10^−4^ for some college education) were independently associated with EEAA. As a sensitivity analysis, we also ran Model 2 in a subgroup of participants who had hypertension and the results were very similar to those from the full sample (Table S4). After adjusting for blood cell proportions, all observed associations for GrimAA remained substantively similar (Table S5). Interestingly, almost all of the associations between lifestyle factors and PhenoAge became stronger with larger effect sizes, except BMI, which stayed the same. Importantly, we observed additional significant effects of gender and education on PhenoAA after adjusting for blood cell proportions.

### 3.4. Interaction between Gender and Lifestyle Factors on DNAm Age Acceleration

**Figure 1:**
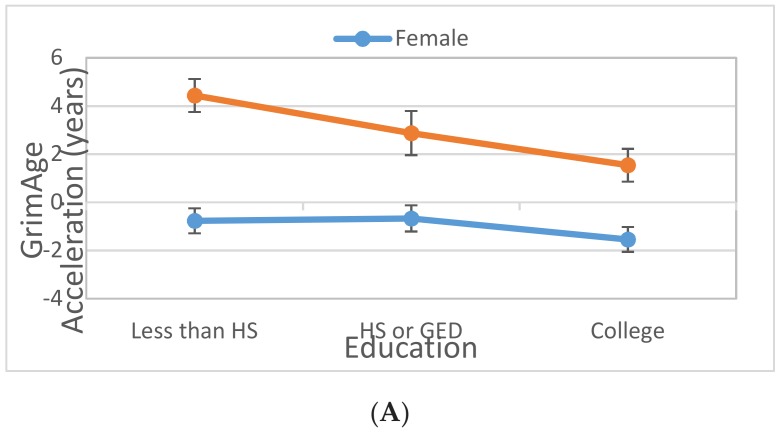

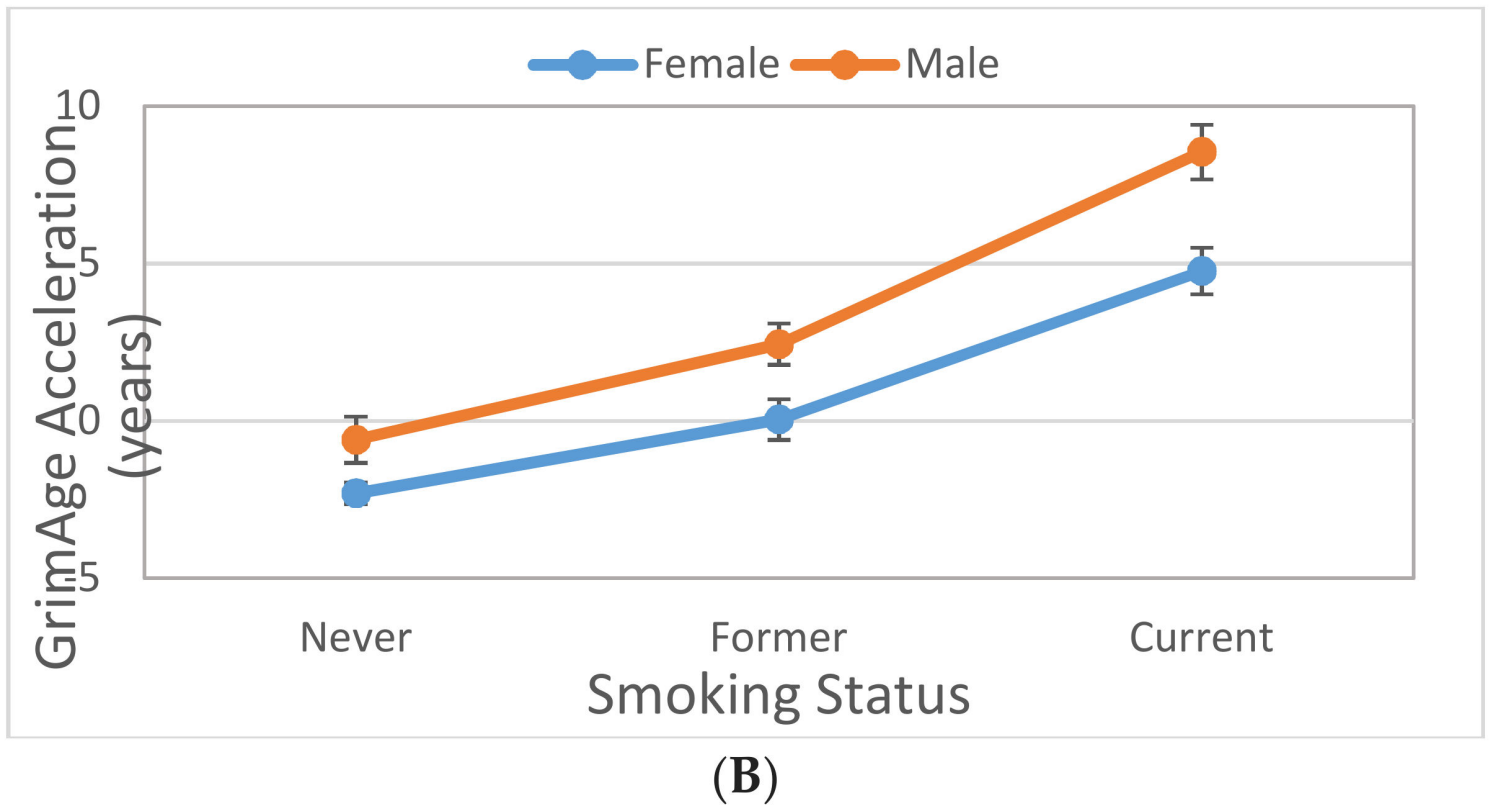
The plots show (**A**) the education effect on GrimAge Acceleration, and (**B**) the smoking effect on GrimAge Accelerations in men (orange line) vs. women (blue line). Each dot represents the least square mean of GrimAge Acceleration for each lifestyle factor-by-gender category, and the bars represent the corresponding confidence intervals for the effect estimate. HS: High School; GED: General Education Development.

There was a significant gender effect on GrimAA, IEAA and EEAA, thus we further assessed if the observed effect of lifestyle (including only those factors significant in Model 2) on GrimAA, IEAA or EEAA varies by gender by adding the corresponding lifestyle-by-gender interaction term to Model 2, one at a time (Model 3). The detected significant interactions are presented in Table 3. We observed strong education-by-gender interactions (*p* = 0.003 for HS/GED × male; *p* = 0.006 for some college × male) as well as a less pronounced smoking-by-gender interaction (*p* = 0.001 for current smoker × male) on GrimAA. As shown in Figure 1, the effect of education on GrimAA was observed in men only (HS vs. no HS: beta = −1.49, CI = (−2.60, −0.38); College vs. no HS: beta = −1.45, CI = (−2.39, −0.51)) and not in women (HS vs. no HS: beta = 0.414, CI = (−0.265, 1.092); College vs. no HS: beta = −0.175, CI = c (−0.849, 0.498)). The effect of current smoking (vs. never smoker) on GrimAA is stronger in men (beta = 8.96, CI = (7.86, 10.05)) compared to women (beta = 6.93, CI = (6.12 7.73)). The effect sizes were similar for former smoker (vs. never smoker) though (men: beta = 2.92, CI = (1.97, 3.88); women: beta = 2.29, CI = (1.58, 2.99). The observed interactions remained significant after adjusting for blood cell proportions (Table S4).

### 3.5. Association between GrimAge Components and Education or Lifestyle Risk Factors

Since education and all lifestyle factors are independently associated with GrimAA, and GrimAge was comprised of DNAm surrogates of eight individual items including seven proteins (DNAmADM, DNAmB2M, DNAmGDF15, DNAmCystatinC, DNAmGDF15, DNAmPAI1, DNAmTIMP1) and the amount of cigarettes smoked (DNAmPACKYRS), we assessed the association between education and lifestyle factors with each individual age-adjusted GrimAge component to further understand the relationship. The multivariable model (Model 2) was used for these analyses, and the results are shown in Table S6. Interestingly, gender was associated with almost all of the GrimAge components except DNAmB2m and DNAmGDF15. Education was not independently associated with any of the GrimAge components. Smoking was not only associated with DNAmPACKYRS as expected, it was also associated with DNAmADM, DNAmB2M, DNAmCystatinC, DNAmGDF15, DNAmPAI1, and DNAmTIMP1, with associations particularly strong for current smoking. Alcohol consumption was associated with DNAmPAI1 and weakly associated with DNAmPACKYRS. BMI was associated with DNAmADM and DNAmLeptin, and to a lesser extent with DNAmCystatinC, DNAmPACKYRS and DNAmTIMP1. After adjusting for blood cell proportions, results stayed similar except for the association between Cystatin C and TIMP1 with gender attenuated (Table S7).

### 3.6. Association between Education or Lifestyle Factors and Longitudinal Change in DNAm Age Acceleration

In a subset of samples whose methylation measurements were collected at both Phase 1 and Phase 2, we assessed whether education and lifestyle factors influence the change in DNAm Age acceleration from Phase 1 to Phase 2 (Model 4). As shown in Table S9, older age (*p* = 0.01) and higher BMI (*p* = 0.028) at Phase 1 increased GrimAA at Phase 2 after adjusting for GrimAA at Phase 1. Similarly, older age (*p* = 0.034) and current smoking (*p* = 0.03) at Phase 1 increased PhenoAA at Phase 2 after adjusting for PhenoAA at Phase 1. No other longitudinal effect was observed between other lifestyle factors and DNAm age measurements. In addition, all of the observed effects were attenuated after adjusting for blood cell proportions (Table S10). This suggests that the longitudinal effects of age, BMI and smoking on DNAm clocks was largely due to changes in blood cell composition, in particular CD4T cells (beta = −9.1, *p* = 0.053 for GrimAA; beta = −18.1, *p* = 0.07 for PhenoAA).

## 4. Discussion

This study evaluated the association between education and lifestyle factors with four important DNAm clocks in a large African-American cohort. It provides evidence of association between multiple DNAm clocks with gender, education, smoking, alcohol consumption and BMI. Specifically, each of these risk factors were all independently associated with GrimAA. Only smoking and BMI were independently associated with PhenoAA. Gender, education and smoking were associated with IEAA, whereas gender and education were also associated with EEAA. The effect of smoking and education on GrimAA varied by gender. The longitudinal analyses suggest that age and BMI continued to increase GrimAA, and that age and current smoking continued to increase PhenoAA after controlling for DNAm Age at baseline. This evidence suggests that different DNAm clocks capture the adverse effects of different environmental stimuli. Consistent with this observation, many DNAm clocks, after adjusting for age, are not highly correlated, which is consistent with other studies [6]. In fact, most of the DNAm clocks are largely comprised of non-overlapping CpG sites. For example, there are only 41 overlapping CpGs between HovarthAge and PhenoAge and only five CpGs that further overlap with HannumAge. Similarly, the association between DNAm clocks and many age-related diseases varies by clocks too, suggesting different DNAm clocks capture different components of cellular aging. All of these suggest that understanding the commonality and differences among those DNAm clocks can help us disentangle the complicated relationship between environment risk factors and age-related chronic diseases. To better understand the biological function of those DNAm clocks, Liu et al. investigated the effect of DNAm clocks on mRNA expressions. Their preliminary results suggest that HannaumAge, HorvathAge and PhenoAge have both shared unique features regarding transcriptional profiles [35]. However, their study did not examine GrimAge. A comprehensive comparative study is needed to understand the functional difference of the CpGs sites that comprise those different DNAm clocks.

### 4.1. Associations with GrimAge Acceleration and Its Components

The most prominent observation of this study is that GrimAA stands out as being independently associated with gender, education, alcohol consumption, smoking, and BMI. These findings are consistent with a previous study in post-menopausal women [6]. However, in the previous study, only the robust correlations between a variety of lifestyle factors and GrimAA were estimated, without controlling for the other lifestyle factors. Thus, it was not clear if the associations they observed were independent of each other. To our knowledge, this was the first study to carefully examine the relationship between education and lifestyle factors with GrimAA simultaneously. Even though gender and smoking were intentionally built into the formula to estimate GrimAge, subsequent analyses on independent GrimAge components suggests that the association between these factors and GrimAA was also due to their relationship with other components.

To better understand GrimAge, we further examined the association between education and lifestyle risk factors and individual DNAm items that comprise the GrimAge. GrimAge includes the methylation signature of seven proteins. Gender, smoking, and BMI were associated with GrimAge items, in particular ADM, Cystatin C, PAI1, and TIMP1. That is not surprising as all those proteins are known to be involved in age-related diseases. Specifically, ADM plays a role regulating fluid and electrolyte homeostasis and is elevated in many cardiorenal diseases [36]. Cystatin-C is a marker of kidney function and also predicts cardiovascular disease [37,38,39]. It is associated with age, male gender, larger weight, and smoking [40]. Elevated PAI1 is a marker of metabolic syndrome associated with obesity and leads to increased risk of heart disease and diabetes [41,42]. TIMP1 is a protein that can promote cell proliferation and is involved in heart failure, diabetes, and cancer [43,44,45]. In addition, B2M and GDF15 were also exclusively associated with smoking. B2M is a clinical marker to help determine myeloma stage [46]. GDF15 is related to inflammation, renal function, atherosclerosis, and cardiovascular mortality [47,48,49,50]. All of these suggest that smoking has a dramatic impact on almost all protein biomarkers that are associated with mortality. The only protein that is not related to smoking is leptin. As expected, leptin is associated with gender and BMI because it is a hormone that inhibits hunger and helps regulate energy balance, and this protein is often elevated in those with obesity [51]. Lastly, alcohol consumption is strongly associated with PAI1, suggesting it affects the metabolic pathway. Interestingly, even though education was associated with GrimAA, it does not appear to influence any GrimAge item significantly. However, college education was consistently (though not significantly) associated with a lower level of DNAm-based surrogates of all proteins. The lack of significance may be due to limited power to detect small effects on each protein surrogate independently, and the effect is only detectable when the components are aggregated together.

### 4.2. Gender and DNAm Clocks

In this study, being male is associated with accelerated GrimAA, IEAA and EEAA. The association between GrimAA and gender is expected because gender was used to estimate GrimAge. However, as pointed out earlier, gender was also associated with several other GrimAge components. The observation that IEAA and EEAA are associated with gender is consistent with previous studies [23]. Similar gender differences in DNAm Age were also reported in children and teenagers [52]. In contrast, a 10-year longitudinal twin study of elderly adults found that age-related changes in methylation at CpG sites were consistent between genders [53]. That is consistent with our observation that gender was not associated with longitudinal change of any of the DNAm clocks in this study. It suggests that gender differences might emerge at an earlier critical developmental stage and then stop influencing the methyolome [54]. However, we cannot rule out the possibility that gender gradually influences the methylome at a very slow rate. The cumulative effect within 5 or 10 years may not be substantial enough to be detected in this or other studies. Nevertheless, all of these suggest that DNAm clocks in blood mirror the gender-specific health disparity regarding life expectancy, and that it is independent of education and many lifestyle risk factors that are often correlated with gender.

### 4.3. Education and DNAm Clocks

In this study, education is significantly associated with GrimAA and EEAA, and to a lesser extent, with IEAA. To be specific, higher education is associated with younger DNAm Age relative to chronological age. Similarly, other studies have observed stronger associations between education and EEAA or HannumAge, and weak or no association with IEAA or HorvathAge [20,22,55]. The association between education and PhenoAA is only marginally significant, which is consistent with a lack of association between education and PhenoAA in African-American women in another study [24]. However, they observed a significant effect in both European Americans and Hispanics. This suggests PhenoAge may capture some ethnic specific component related to education. Thus, the lack of association between PhenoAA and education in our study may be due to the fact that this is an African-American cohort. However, the association between PhenoAA and education becomes significant after adjusting for blood cell proportions, suggesting that some intrinsic component of PhenoAge is influenced by education. Future analyses in large African-American cohorts are needed to confirm our finding.

The most interesting finding with respect to education associations with DNAm clocks is the relationship between education and GrimAA, which varies by gender with a significant association observed only in men after adjusting for all other lifestyle risk factors. Contrary to that, Lu et al. looked at the bivariate correlation between education and GrimAA in women and found a small significant correlation (r = −0.09) [6]. It was undetermined if the correlation would remain after adjusting for other lifestyle factors. To our knowledge, no other study has ever examined the association between education and GrimAA. Thus, more studies investigating education and GrimAA are needed to validate our finding. Importantly, our study suggests that education affects DNAm Age through some mechanism that is independent of common lifestyle risk factors. Other studies suggest that financial strain might be an important risk factor that contributes to the association between education and DNAm clocks [56,57].

### 4.4. Smoking and DNAm Clocks

Smoking is known to have a dramatic impact on DNA methylation [58,59,60]. This is not surprising given the fact that smokers on average lose 10 years of life expectancy compared to non-smokers. However, consistent with our observation, neither IEAA nor EEAA seems to capture much of the toxic effects of smoking [20]. Compared to that, PhenoAge has been shown to do a better job capturing adverse effects from smoking in this study and others [5,22]. GrimAge, by definition, incorporated CpGs that are surrogate markers of smoking. However, as discussed earlier, the toxic effect of smoking was not only captured by DNAm-based surrogates of smoking, but also captured by DNAm-based surrogates of many proteins, including ADM, B2M, CystatinC, GDF15, PAI1 and TIMP1. This suggests that smoking has a dramatic impact on various biological pathways. Our study also found that the association between smoking and GrimAA varies by gender as the association is stronger in men than women. Interestingly, previous literature suggests that smoking has gender-specific effects on many health conditions, including inflammation, lung function, neuroendocrine function, and many cancers [61,62,63,64]. The gender-specific effect of smoking on DNA methylation may be a potential underlying cellular mechanism mediating the gender-specific effect of smoking on health outcomes.

### 4.5. BMI and DNAm Clocks

Similar to smoking, BMI is known to be associated with methylation at many CpG sites throughout the genome [65,66,67]. In this study, it was associated with PhenoAA and GrimAA, but not IEAA or EEAA. Previous studies have shown an association between BMI and EEAA/IEAA [20,22]. However, it seems that the association may vary by ethnicity and was robust only in Whites. Future studies in non-European populations are required to understand the potential heterogeneity across ethnicities. In comparison, the relationship of BMI with PhenoAA and GrimAA are consistent with previous observations [6,22]. Since BMI represents an internal physical environment, the causality between BMI and changes in methylation could happen in both directions. However, recent studies suggest that alterations in DNA methylation are more likely to be the consequence of obesity [66,67,68]. This is also supported by intergenerational analysis [69,70]. Consistent with that hypothesis, we observed an association between baseline BMI and longitudinal change in GrimAA. This evidence supports a causal role of BMI on DNAm Age.

### 4.6. Alcohol Consumption and DNAm Clocks

Alcohol consumption was not associated with most of the DNAm clocks, with the exception of GrimAA, which was positively associated. There has been inconsistent evidence regarding the relationship between alcohol consumption and DNAm Age. A recent large-scale meta-analyses revealed an association between alcohol consumption and PhenoAA, but not with HorvathAge or HannumAge [22]. Other studies suggest a nonlinear relationship between alcohol consumption and DNAm Age [20,71]. On the other hand, a previous study also suggests that the association between alcohol consumption and DNAm age varies by ethnicity as they observed a significant association between drinking status and EEAA in Caucasian and Hispanic participants, but not African or Asian participants [20]. Also, it is widely known that alcohol metabolism is different among racial groups [72]. Thus, it is possible that alcohol influences the methylome differentially across ethnicities [73]. More studies on multiethnic cohorts are needed to better understand this relationship.

### 4.7. Physical Activity and DNAm Clocks

Even though we did not detect an association between physical activity and DNAm clocks, the direction of effect is consistent with what we would expect. Lack of significance may be due to the fact that the physical activity was collected at Phase 2, which may not be a good surrogate for physical activity at Phase 1. However, consistent with our observation, a few other studies, including a recent large scale meta-analysis on more than 16K participants of European ancestry, also failed to find an association between physical activity and DNAm age [5,20,22,57,74,75]. However, one small study reported negative association between 7-day step count and EEAA in older adults at age of 79 [76]. The results of that study may be subject to selection bias and not generalizable to the general population. Lu et al. reported a significant correlation between physical activity and GrimAge in non-Hispanic white women, but not in African-American or Hispanic women [6], suggesting that the relationship between physical activity and DNAm clocks may vary by ethnicity. Future studies with better physical activity measurement in large multi-ethnic cohorts are needed to better understand the complicated interplay between physical activity and DNAm clocks.

### 4.8. Lifestyle Factors and Longitudinal Change in DNAm Age Acceleration

Using powerful longitudinal design, we observed an association between chronological age and change in PhenoAA and GrimAA over an approximate 5-year time span. This suggests that the two DNAm clocks run (or age) faster at older ages. However, another study suggests HorvathAge and HannumAge tend to age slower at older ages [1]. However, this study investigated HorvathAge and HannumAge, rather than PhenoAA and GrimAA, and was conducted in a European population. Twin studies suggest that environmental influences on DNA methylation increase over time [77,78]. It is also possible that the age effect we observed here was due to some emerging unknown environmental influence. In addition, we found that higher BMI was associated with increased GrimAA and current smoking was associated with increased PhenoAA. This suggests a causal and immediate effect of BMI and current smoking on the corresponding DNAm clocks. So far there have been very few studies examining the relationship between smoking with longitudinal change in methylation, but the few that exist have all found that smoking does cause long-term changes at some methylation sites [60,79]. Similar findings have emerged for BMI [68,80]. Nevertheless, our study suggests that smoking cessation and weight reduction are likely to yield immediate beneficial effects on health aging. In our study, all of the longitudinal effects were attenuated after adjusting for blood cell proportions, suggesting that the associations were largely due to changes in the cell counts. Lastly, our sample size for longitudinal analyses was fairly small, thus it is likely that we did not have enough power to detect small effect sizes that may be conferred by other lifestyle risk factors. Large-scale longitudinal studies are the next very important step to advance our understanding of the causal relationships and mechanisms underlying lifestyle risk factors and the methylome.

### 4.9. Strengths and Limitations

This study has some limitations. First, this is a predominately hypertensive cohort, and the conclusions drawn from this study may not be generalizable to non-hypertensive populations. Second, GENOA did not collect other lifestyle risk factors, such as diet, which may confound some of the results. Third, the sample size for the longitudinal analyses was relatively small which may limit our power to detect associations. Regardless of these limitations, this is the first study, to the best of our knowledge, that comprehensively examined the association of education and many lifestyle risk factors with multiple DNAm clocks, including the new clock GrimAge, in a large, gender-balanced African-American cohort. This allowed us to examine the marginal effects of education and lifestyle factors on DNAm clocks as well as potential interaction with gender in African-Americans. The other unique contribution of this study is its longitudinal component which allowed us to assess the temporal relationship between lifestyle factors and change in DNAm clocks.

## 5. Conclusions

Overall, our study indicates that many DNAm clocks were associated with education and a variety of lifestyle risk factors in African Americans. However, the strength of the associations varies by clock, which suggests different DNAm clocks capture the adverse effects from different external environmental stimuli. Among all of the clocks we examined, GrimAge stands out as being influenced by all lifestyle risk factors in the expected direction. The above evidence, along with the fact that GrimAge performs better at predicting age-related disease and mortality, suggests that it may be a promising DNAm clock that best mirrors the relationship between environmental risk factors and healthy aging. Future studies focusing on GrimAge and related items are needed to advance our understanding about cellular aging.

## Figures and Tables

**Table 1:** Descriptive statistics of study participants from the Genetic Epidemiology Network of Arteriopathy (GENOA).

	Cross-Sectional (N = 1100)	Longitudinal (N = 266)		
Variable Name	Mean (SD) or N (%)	Phase 1 Mean (SD) or N (%)	Phase 2 Mean (SD) or N (%) ^1^	Pearson r or Kappa ^2^
HorvathAge (years)	53.89 (9.98)	51.19 (9.18)	55.12 (9.19) ***	0.919
HannumAge (years)	47.71 (10.75)	44.27 (9.75)	49.14 (10.02) ***	0.960
PhenoAge (years)	44.22 (12.66)	40.21 (11.62)	44.68 (11.69) ***	0.922
GrimAge (years)	54.31 (9.49)	52.00 (9.09)	55.44 (9.15) ***	0.972
IEAA (years)	0.15 (4.79)	−0.01 (4.77)	−0.46 (5.10) *	0.748
EEAA (years)	0.27 (5.85)	−0.36 (5.54)	−1.02 (6.52) **	0.852
PhenoAA (years)	0.38 (7.17)	−0.54 (6.81)	−1.46 (6.27) **	0.764
GrimAA (years)	0.11 (4.95)	0.23 (4.56)	−0.58 (4.63) ***	0.911
Chronological Age (years)	57.05 (10.48)	53.97 (9.77)	59.42 (9.35) ***	0.994
Gender (male)	319 (29.00%)	77 (28.95%)		
Education				
Less than HS	374 (34.00%)	80 (30.08%)		
HS/GED	292 (26.55%)	71 (26.69%)		
At least some college	434 (39.45%)	115 (43.23%)		
Smoking				
Never	666 (60.55%)	160 (60.15%)		
Former smoker	255 (23.18%)	63 (23.68%)		
Current smoker	179 (16.27%)	43 (16.17%)		
Continuous drinks/week	0.67 (2.68)	0.71 (2.82)	0.52 (1.69)	0.491
Physical activity (hrs/day)	1.07 (1.57)		1.14 (1.42)	
Body Mass Index (kg/m^2^)	31.20 (6.48)	31.53 (6.69)	32.04 (6.83) ***	0.946

IEAA: intrinsic epigenetic age acceleration; EEAA: extrinsic epigenetic age acceleration; PhenoAA: DNAm PhenoAge acceleration; GrimAA: DNAm GrimAge acceleration; HS/GED: High School/General Education Development. ^1^ Paired *t*-tests (for continuous variables) or Bowker’s test of symmetry (for categorical variables) were conducted to compare the distribution at Phase 1 vs. Phase 2. ^2^ Correlation coefficient between phase 1 and phase 2 measurements. *** *p*-value ≤ 0.001, ** *p*-value ≤ 0.01, * *p*-value ≤ 0.05.

**Table 2:** Associations of DNAm Age acceleration with education and lifestyle factors using multivariable models (Model 2).

	GrimAA		PhenoAA		IEAA		EEAA	
	Beta	*p*-Value	Beta	*p*-Value	Beta	*p*-Value	Beta	*p*-Value
Gender (male)	2.410	**1.94 × 10^−17^**	−0.834	0.106	1.038	**0.003**	2.405	**1.94 × 10^−8^**
Education								
HS/GED	−0.098	0.745	−0.812	0.153	−0.669	0.081	−1.016	**0.028**
At least some college	−0.605	**0.041**	−1.107	0.051	−0.743	**0.050**	−1.784	**1.02 × 10^−4^**
Smoking								
Former smoker	2.337	**2.67 × 10^−15^**	1.317	**0.015**	0.948	**0.009**	0.039	0.928
Current smoker	7.618	**4.98 × 10^−81^**	2.135	**0.001**	0.045	0.915	0.672	0.191
Continuous ln(drinks/week)	0.455	**0.033**	0.561	0.159	0.376	0.164	−0.044	0.891
BMI	0.040	**0.029**	0.080	**0.022**	0.038	0.112	0.035	0.216

IEAA: intrinsic epigenetic age acceleration; EEAA: extrinsic epigenetic age acceleration; GrimAA: DNAm GrimAge acceleration; PhenoAA: DNAm PhenoAge acceleration; HS/GED: High School/General Education Development. Model: DNAm Age acceleration~ gender + education + age + smoking + alcohol consumption + BMI. Beta is the regression coefficient of the respective variable from the regression model as stated above. Significant *p* values (<0.05) are bold.

**Table 3:** Interactions between lifestyle risk factors and gender on GrimAA (Model 3).

	GrimAA Interaction Model ^1^		GrimAA Interaction Model ^2^		GrimAA Interaction Model ^3^	
	Beta	*p*-Value	Beta	*p*-Value	Beta	*p*-Value
Gender (male)	3.450	**8.79 × 10^−16^**	1.684	**2.43 × 10^−5^**	2.698	**4.75 × 10^−7^**
Education						
HS/GED	0.439	0.205	−0.108	0.720	0.414	0.231
At least some college	−0.114	0.739	−0.592	**0.045**	−0.175	0.610
Smoking						
Former smoker	2.365	**1.06 × 10^−15^**	2.213	**1.42 × 10^−9^**	2.287	**4.08 × 10^−10^**
Current smoker	7.622	**2.34 × 10^−81^**	6.877	**1.46 × 10^−51^**	6.928	**2.57 × 10^−52^**
Continuous ln (drinks/week)	0.462	**0.031**	0.372	0.083	0.380	0.077
BMI	0.045	**0.016**	0.041	**0.028**	0.044	**0.017**
Gender (male)*Education						
HS/GED	−1.929	**0.003**			−1.899	**0.003**
At least some college	−1.513	**0.006**			−1.272	**0.022**
Gender (male)*Smoking						
Former smoker			0.786	0.189	0.636	0.290
Current smoker			2.170	**0.001**	2.026	**0.003**

GrimAA: DNAm GrimAge acceleration; HS/GED: High School/General Education Development. ^1^ Model: GrimAA~ age + alcohol consumption + BMI + smoking + gender*education. ^2^ Model: GrimAA~ age + alcohol consumption + BMI + education + gender*smoking. ^3^ Model: GrimAA~ age + alcohol consumption + BMI + gender*education + gender*smoking. Beta is the regression coefficient of the respective variable from the regression model as stated above. Significant *p* values (<0.05) are bolded. No other gender interactions with GrimAA or other age acceleration measures were significant.

## Supplementary Materials

The following are available online at https://www.mdpi.com/1660-4601/16/17/3141/s1, Figure S1: Scatterplots of DNAm Age estimators vs. chronological age for participants in the Genetic Epidemiology Network of Arteriopathy (GENOA, Phase 1); Table S1: Pairwise correlation between DNAm Age estimates, DNAm Age acceleration estimates and chronological age; Table S2: Association of DNAm Age acceleration with education and lifestyle factors (Model 1); Table S3: Association of DNAm Age acceleration with education and lifestyle factors (including physical activity) using multivariable models (Model 2); Table S4: Association of DNAm Age acceleration with education and lifestyle factors using multivariable models in hypertensive participants (Model 2, N = 771); Table S5: Association of DNAm Age acceleration with education and lifestyle factors adjusting for blood cell proportions; Table S6: Interaction between lifestyle risk factors and gender on GrimAA, adjusting for blood cell proportions; Table S7: Associations of GrimAge components with education and lifestyle factors (Model 2); Table S8: Associations of DNAm GrimAge components with demographic and lifestyle factors, adjusting for blood cell proportions; Table S9: Association between education and lifestyle factors on longitudinal change in DNAm Age acceleration (Model 4); Table S10: Associations between education and lifestyle factors and longitudinal change of DNAm Age acceleration, adjusting for blood cell proportions.
