# Supplementary material for: Education and Lifestyle Factors Are Associated with DNA Methylation Clocks in Older African Americans
Source: Int J Environ Res Public Health. 2019 Aug 28;16(17):3141. doi: 10.3390/ijerph16173141 (PMC6747433; doi:10.3390/ijerph16173141)
Supplement: Supplementary file 1 [file ijerph-16-03141-s001.pdf]

Table S1: Pairwise correlation between DNAm age estimates, DNAm Age acceleration estimates and chronological age.

Pairwise Correlation	HorvathAge	HannumAge	PhenoAge	GrimAge	IEAA	EEAA	PhenoAA	GrimAA
Chronological Age	0.863***	0.898***	0.824***	0.855***	0.020	0.002	−0.005	−0.026
HorvathAge		0.899***	0.843***	0.781***	0.499***	0.27***	0.229***	0.06*
HannumAge			0.853***	0.812***	0.228***	0.424***	0.196***	0.062*
PhenoAge				0.797***	0.264***	0.283***	0.563***	0.158***
GrimAge					0.109***	0.134***	0.16***	0.497***
IEAA						0.417***	0.436***	0.175***
EEAA							0.496***	0.255***
PhenoAA								0.316***

IEAA: intrinsic epigenetic age acceleration; EEAA: extrinsic epigenetic age acceleration; GrimAA: DNAm GrimAge acceleration; PhenoAA: DNAm PhenoAge acceleration.

P-values were calculated from Pearson's correlation tests.

*** p-value ≤ 0.001, ** p-value ≤ 0.01, * p-value ≤ 0.05.

Table S2: Associations of DNAm Age acceleration with education and lifestyle factors (Model 1)

	GrimAA		PhenoAA		IEAA		EEAA	
	beta	p-value	beta	p-value	beta	p-value	beta	p-value
Gender (male)	4.044	**1.69×10^{-35}**	–0.211	0.647	1.331	**2.16×10^{-5}**	2.449	**1.26×10^{-10}**
Education								
HS/GED	–0.696	0.060	–1.049	0.065	–0.754	**0.049**	–1.080	**0.019**
At least some college	–1.780	**7.56×10^{-7}**	–1.600	**0.004**	–0.879	**0.018**	–1.912	**2.19×10^{-5}**
Smoking								
Former smoker	2.474	**5.30×10^{-17}**	1.541	**0.004**	1.090	**0.003**	0.243	0.578
Current smoker	7.793	**1.16×10^{-88}**	2.325	**1.42×10^{-4}**	0.213	0.603	0.886	0.074
Continuous ln(drinks/week)	1.535	**2.76×10^{-9}**	0.810	**0.038**	0.368	0.164	0.084	0.792
Physical activity	–0.119	0.185	–0.132	0.337	–0.027	0.765	–0.207	0.064
BMI	–0.013	0.552	0.070	**0.044**	0.044	0.060	0.039	0.169

IEAA: intrinsic epigenetic age acceleration; EEAA: extrinsic epigenetic age acceleration; GrimAA: DNAm GrimAge acceleration; PhenoAA: DNAm PhenoAge acceleration; HS/GED: High School/General Education Development.

Model: DNAm Age acceleration~ age + gender + lifestyle risk factor (one at a time)

Beta is the regression coefficient of the respective variable from the regression model as stated above.

Significant p values (<0.05) are bolded.

Table S3: Associations of DNAm Age acceleration with education and lifestyle factors (including physical activity) using multivariable models (Model 2)

	**GrimAA**		**PhenoAA**		**IEAA**		**EEAA**	
	**beta**	**p-value**	**beta**	**p-value**	**beta**	**p-value**	**beta**	**p-value**
Gender (male)	2.497	**3.47×10^{-8}**	–0.826	0.113	1.054	**0.003**	2.504	**6.66×10^{-9}**
Education								
HS/GED	–0.056	0.854	–0.787	0.169	–0.683	0.077	–1.033	**0.027**
At least some college	–0.589	**0.049**	–1.028	0.073	–0.791	**0.039**	–1.723	**2.20×10^{-4}**
Smoking								
Former smoker	2.324	**4.56×10^{-15}**	1.355	**0.012**	0.981	**0.007**	0.060	0.892
Current smoker	7.513	**2.71×10^{-78}**	2.039	**0.001**	0.106	0.805	0.709	0.174
Continuous								
ln(drinks/week)	0.491	**0.022**	0.594	0.139	0.402	0.138	-0.007	0.983
BMI	0.043	**0.021**	0.088	**0.014**	0.039	0.103	0.035	0.222
Physical activity (hrs/day)	–0.047	0.522	–0.073	0.594	0.006	0.951	-0.141	0.208

IEAA: intrinsic epigenetic age acceleration; EEAA: extrinsic epigenetic age acceleration; GrimAA: DNAm GrimAge acceleration; PhenoAA: DNAm PhenoAge acceleration; HS/GED: High School/General Education Development.

Model: DNAm Age acceleration~ gender + education + age + smoking + alcohol consumption + BMI + physical activity

Beta is the regression coefficient of the respective variable from the regression model as stated above

Significant p values (<0.05) are bold

Table S4: Associations of DNAm Age acceleration with education and lifestyle factors using multivariable models in hypertensive participants (Model 2, N=771)

	**GrimAA**		**PhenoAA**		**IEAA**		**EEAA**	
	**beta**	**p-value**	**beta**	**p-value**	**beta**	**p-value**	**beta**	**p-value**
Gender (male)	2.338	**1.60×10^{-11}**	−0.512	0.440	1.519	**0.001**	2.708	**7.87×10^{-7}**
Education								
HS/GED	0.026	0.941	−1.059	0.125	−0.942	**0.041**	−1.177	**0.036**
At least some college	−0.222	0.519	−1.650	**0.017**	−1.357	**0.003**	−2.227	**6.64×10^{-5}**
Smoking								
Former smoker	2.512	**5.62×10^{-13}**	1.425	**0.032**	1.177	**0.008**	0.428	0.426
Current smoker	7.998	**3.27×10^{-54}**	2.384	**0.004**	−0.077	0.888	0.675	0.311
Continuous								
ln(drinks/week)	0.390	0.146	0.691	0.193	0.084	0.813	−0.445	0.303
BMI	0.041	0.064	0.091	**0.039**	0.008	0.786	0.032	0.374

IEAA: intrinsic epigenetic age acceleration; EEAA: extrinsic epigenetic age acceleration; GrimAA: DNAm GrimAge acceleration; PhenoAA: DNAm PhenoAge acceleration; HS/GED: High School/General Education Development.

Model: DNAm Age acceleration~ gender + education + age + smoking + alcohol consumption + BMI

Beta is the regression coefficient of the respective variable from the regression model as stated above.

Significant p values (< 0.05) are bold.

Table S5: Association of DNAm Age acceleration with education and lifestyle factors adjusting for blood cell proportions

	GrimAA		PhenoAA	
	beta	p-value	beta	p-value
Gender (male)	2.018	**1.27×10^{-13}**	−1.879	**1.64×10^{-4}**
Education				
HS/GED	−0.265	0.351	−0.964	0.069
At least some college	−0.821	**0.003**	−1.321	**0.013**
Smoking				
Former smoker	2.445	**2.24×10^{-18}**	1.609	**0.001**
Current smoker	7.762	**3.36×10^{-88}**	2.748	**5.88×10^{-6}**
Continuous ln(drinks/week)	0.409	**0.043**	0.574	0.124
BMI	0.040	**0.023**	0.082	**0.013**

GrimAA: DNAm GrimAge acceleration; PhenoAA: DNAm PhenoAge acceleration; HS/GED: High School/General Education Development.

Model: DNAm Age acceleration~ age + gender + education + smoking + alcohol consumption + BMI + CD8T + CD4T + NK + Bcell + Mono

Beta is the regression coefficient of the respective variable from the regression model as stated above.

Significant p values (<0.05) are bolded.

Table S6: Interaction between lifestyle risk factors and gender on GrimAA, adjusting for blood cell proportions

	GrimAA Interaction Model[1]		GrimAA Interaction Model[2]		GrimAA Interaction Model[3]	
	beta	p-value	beta	p-value	beta	p-value
Gender (male)	2.897	**8.97 × 10^{-13}**	1.359	**3.19 × 10^{-4}**	2.202	**1.25×10^{-5}**
Education						
HS/GED	0.187	0.566	–0.268	0.344	0.170	0.601
At least some college	–0.401	0.215	–0.803	**0.004**	–0.454	0.161
Smoking						
Former smoker	2.469	**9.43× 10^{-19}**	2.419	**2.48 × 10^{-12}**	2.481	**7.53 × 10^{-13}**
Current smoker	7.769	**2.02×10^{-88}**	6.962	**8.60 × 10^{-57}**	7.010	**1.89 × 10^{-57}**
Continuous ln(drinks/week)	0.413	**0.040**	0.319	0.116	0.322	0.111
BMI	0.043	**0.013**	0.040	**0.021**	0.043	**0.013**
Gender (male)*Education						
HS/GED	–1.605	**0.009**			–1.579	**0.009**
At least some college	–1.290	**0.012**			–1.060	**0.041**
Gender (male)*Smoking						
Former smoker			0.548	0.329	0.424	0.453
Current smoker			2.254	**3.26 × 10^{-4}**	2.135	**0.001**

GrimAA: DNAm GrimAge acceleration; HS/GED: High School/General Education Development.

[1]Model: GrimAA~ age + alcohol consumption + BMI + smoking + gender*education + CD8T + CD4T + NK + Bcell + Mono

[2]Model: GrimAA~ age + alcohol consumption + BMI + education + gender*smoking + CD8T + CD4T + NK + Bcell + Mono

[3]Model: GrimAA~ age + alcohol consumption + BMI + gender*education + gender*smoking + CD8T + CD4T + NK + Bcell + Mono

Beta is the regression coefficient of the respective variable from the regression model as stated above.

Significant p values (<0.05) are bolded.

No other interactions between sex and lifestyle factors on GrimAA or other age acceleration measures were significant.

Table S7: Associations of GrimAge components with education and lifestyle risk factors (Model 2)

	DNAmADM		DNAmB2M		DNAmCystatinC		DNAmGDF15	
	beta	p-value	beta	p-value	beta	p-value	beta	p-value
Gender (male)	–24.430	**5.03 × 10^{-68}**	–10371.390	0.309	5642.568	**0.001**	15.123	0.075
Education								
HS/GED	0.280	0.834	–13470.036	0.234	143.005	0.938	5.113	0.584
At least some college	–0.216	0.869	–17908.017	0.125	–3026.581	0.093	–6.152	0.501
Smoking								
Former smoker	0.145	0.909	8278.261	0.434	–1508.296	0.386	22.241	**0.013**
Current smoker	3.299	**0.027**	26030.929	**0.038**	4489.474	**0.028**	73.695	**4.41 × 10^{-12}**
Continuous ln(drinks/week)	1.015	0.284	9866.188	0.205	241.873	0.851	7.462	0.259
BMI	0.384	**3.86 × 10^{-6}**	168.349	0.812	269.670	**0.017**	0.449	0.435

	DNAmLeptin		DNAmPACKYRS		DNAmPAI1		DNAmTIMP1	
	beta	p-value	beta	p-value	beta	p-value	beta	p-value
Gender (male)	–7239.797	**1.47 × 10^{-228}**	4.532	**1.71 × 10^{-12}**	993.852	**5.78 × 10^{-8}**	151.862	**0.041**
Education								
HS/GED	–101.940	0.481	0.437	0.526	–71.098	0.720	–72.823	0.373
At least some college	–106.616	0.453	–0.890	0.183	–56.631	0.769	–134.668	0.093
Smoking								
Former smoker	70.520	0.609	9.007	**6.12 × 10^{-37}**	488.310	**0.010**	–68.760	0.377
Current smoker	98.138	0.543	26.131	**3.52 × 10^{-138}**	727.241	**0.001**	208.182	**0.023**
Continuous ln(drinks/week)	28.087	0.784	0.975	**0.047**	780.226	**4.78 × 10^{-8}**	–23.520	0.684
BMI	42.474	**2.34 × 10^{-6}**	–0.119	**0.005**	116.191	**3.53 × 10^{-20}**	9.956	**0.048**

DNAmADM: DNAm surrogate of adrenomedullin (ADM); DNAmB2M: DNAm surrogate of beta-2 microglobulin (B2M), DNAmGDF15: DNAm surrogate of growth differentiation factor 15 (GDF15); DNAmCystatinC: DNAm surrogate of Cystatin C (CystatinC); DNAmLeptin: DNAm surrogate of leptin (Leption); DNAmPAI1: DNAm surrogate of plasminogen activation inhibitor 1 (PAI1); DNAmTIMP1: DNAm surrogate of Issue inhibitor metalloproteinase 1 (TIMP1); DNAmPACKYRS: DNAm surrogate of the amount of cigarettes smoked (PACKYRS); HS/GED: High School/General Education Development.

Model: DNAm GrimAge components ~ age + gender + education + smoking + alcohol consumption + BMI + CD8T + CD4T + NK + Bcell + Mono

Beta is the regression coefficient of the respective variable from the regression model as stated above.

Significant p values (<0.05) are bolded.

Table S8: Associations of DNAm GrimAge components with demographic and lifestyle factors, adjusting for blood cell proportions

	DNAmADM		DNAmB2M		DNAmCystatinC		DNAmGDF15	
	beta	p-value	beta	p-value	beta	p-value	beta	p-value
Gender (male)	–24.903	**1.97×10^{-69}**	–19758.128	0.060	2155.375	0.175	9.652	0.267
Education								
HS/GED	–0.339	0.795	–14150.526	0.208	–310.759	0.854	4.524	0.626
At least some college	–1.033	0.420	–18218.918	0.117	–3764.063	**0.025**	–7.352	0.419
Smoking								
Former smoker	0.445	0.721	8330.040	0.430	–575.282	0.721	24.216	**0.006**
Current smoker	3.207	**0.031**	30438.674	**0.017**	6426.126	**0.001**	76.924	**1.09×10^{-12}**
Continuous ln(drinks/week)	0.849	0.360	10392.573	0.183	81.129	0.946	8.213	0.214
BMI	0.386	**1.85×10^{-6}**	215.658	0.759	287.189	**0.006**	0.506	0.375

	DNAmLeptin		DNAmPACKYRS		DNAmPAI1		DNAmTIMP1	
	beta	p-value	beta	p-value	beta	p-value	beta	p-value
Gender (male)	–7211.810	**1.84×10^{-220}**	4.267	**8.07×10^{-11}**	908.208	**9.96×10^{-7}**	48.579	0.502
Education								
HS/GED	–75.093	0.604	0.208	0.763	-133.675	0.495	–114.991	0.137
At least some college	–80.669	0.570	–1.158	0.082	-137.007	0.473	–190.078	**0.013**
Smoking								
Former smoker	62.129	0.652	9.057	**1.97×10^{-37}**	560.518	**0.003**	–44.452	0.546
Current smoker	93.209	0.572	26.167	**2.29×10^{-135}**	794.468	**3.98×10^{-4}**	241.527	**0.006**
Continuous ln(drinks/week)	10.989	0.915	0.900	0.067	738.914	**1.75×10^{-7}**	–34.049	0.535
BMI	42.915	**1.78×10^{-6}**	–0.123	**0.004**	115.526	**1.87×10^{-20}**	10.085	**0.034**

DNAmADM: DNAm surrogate of adrenomedullin (ADM); DNAmB2M: DNAm surrogate of beta-2 microglobulin (B2M), DNAmGDF15: DNAm surrogate of growth differentiation factor 15 (GDF15); DNAmCystatinC: DNAm surrogate of Cystatin C (CystatinC); DNAmLeptin: DNAm surrogate of leptin (Leption); DNAmPAI1: DNAm surrogate of plasminogen activation inhibitor 1 (PAI1); DNAmTIMP1: DNAm surrogate of Issue inhibitor metalloproteinase 1 (TIMP1); DNAmPACKYRS: DNAm surrogate of the amount of cigarettes smoked (PACKYRS); HS/GED: High School/General Education Development.

Model: DNAm GrimAge components ~ age + gender + education + smoking + alcohol consumption + BMI + CD8T + CD4T + NK + Bcell + Mono

Beta is the regression coefficient of the respective variable from the regression model as stated above.

Significant p values (<0.05) are bolded.

Table S9: Associations between education and lifestyle factors on longitudinal change in DNAm Age acceleration (Model 4)

	GrimAA		PhenoAA		IEAA		EEAA	
	**beta**	**p-value**	**beta**	**p-value**	**beta**	**p-value**	**beta**	**p-value**
Age	0.050	**0.010**	0.081	**0.034**	–0.006	0.820	0.050	0.050
Gender (male)	0.692	0.057	1.448	0.052	0.381	0.500	1.184	0.692
Education								
HS/GED	–0.078	0.819	0.121	0.866	–0.382	0.545	–0.512	–0.078
At least some college	–0.106	0.745	–0.530	0.446	0.320	0.590	–0.696	–0.106
Smoking								
Former smoker	0.295	0.397	0.154	0.825	–0.409	0.510	–0.095	0.295
Current smoker	0.636	0.188	2.029	**0.031**	–0.530	0.440	0.404	0.636
Continuous ln(drinks/week)	–0.090	0.737	–0.469	0.426	0.243	0.626	–0.877	–0.090
BMI	0.050	**0.028**	0.079	0.082	0.047	0.207	0.026	0.050

IEAA: intrinsic epigenetic age acceleration; EEAA: extrinsic epigenetic age acceleration; GrimAA: DNAm GrimAge acceleration; PhenoAA: DNAm PhenoAge acceleration; HS/GED: High School/General Education Development.

Model: DNAm Age acceleration2 ~ DNAm Age acceleration1 + gender + education1 + Age1 + smoking1 + alcohol consumption1 + BMI1 + Δtime + ΔBMI + Δalcohol consumption. People whose smoking status changed between phases were excluded.

Beta is the regression coefficient of the respective variable from the regression model as stated above.

Significant p values ($p<0.05$) are bolded.

Table S10: Associations between education and lifestyle factors and longitudinal change of DNAm Age acceleration, adjusting for blood cell proportions

	GrimAA		PhenoAA	
	beta	p-value	beta	p-value
Age	0.014	0.359	0.019	0.568
Gender (male)	0.214	0.480	0.764	0.304
Education				
HS/GED	–0.220	0.487	0.034	0.961
At least some college	–0.100	0.726	–0.386	0.583
Smoking				
Former smoker	–0.014	0.963	–0.025	0.971
Current smoker	0.192	0.637	1.890	0.109
Continuous ln(drinks/week)	0.047	0.842	–0.323	0.581
BMI	0.027	0.211	0.042	0.353

GrimAA: DNAm GrimAge acceleration; PhenoAA: DNAm PhenoAge acceleration; HS/GED: High School/General Education Development.
Model: DNAm Age $acceleration_2$ ~ DNAm Age $acceleration_1$ + gender + $education_1$ + Age_1 + $smoking_1$ + alcohol $consumption_1$ + BMI_1 + Δtime + ΔBMI + Δalcohol consumption + ΔCD8T + ΔCD4T + ΔNK + ΔBcell + ΔMono. People whose smoking status changed were excluded.
Beta is the regression coefficient of the respective variable from the regression model as stated above.
Significant p values (p<0.05) are bolded.

Figure S1: Scatterplots of DNAm Age estimators vs. chronological age for participants in the Genetic Epidemiology Network of Arteriopathy (GENOA, Phase 1). Chronological age is on the X axis, and the Y axis is (A) HorvathAge; (B) HannumAge; (C) PhenoAge; (D) GrimAge.

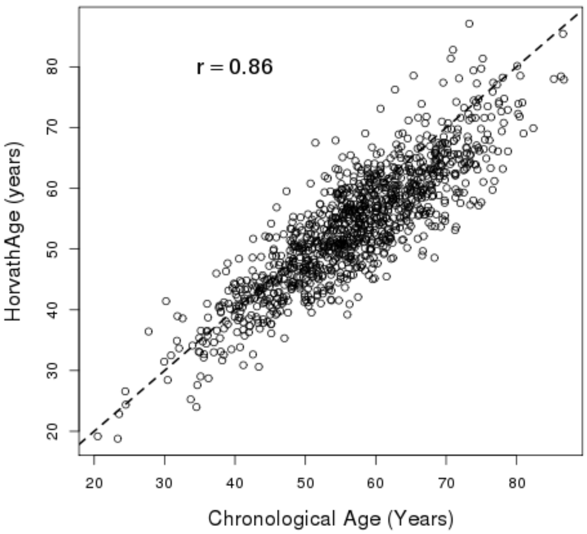

(a)

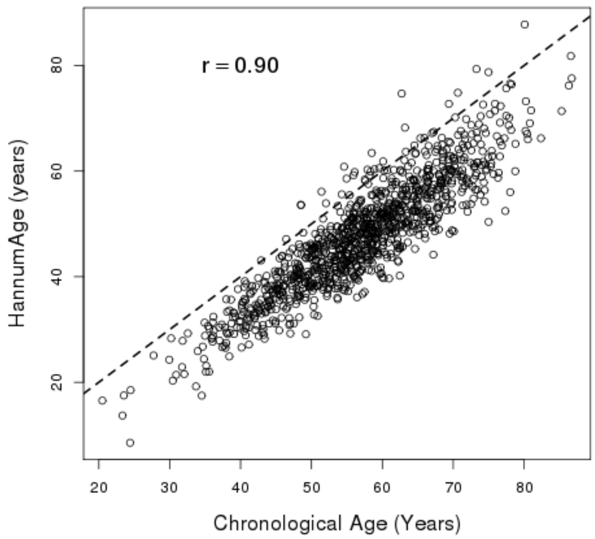

(b)

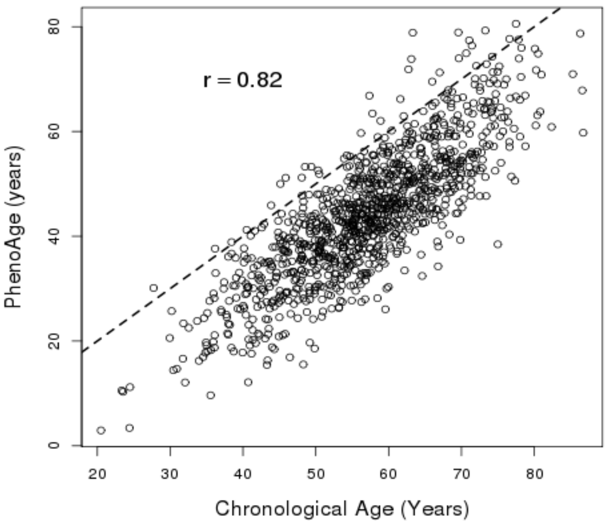

(c)

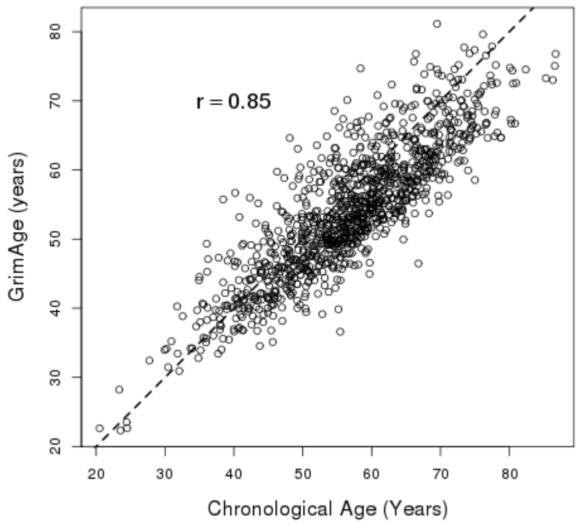

(d)
